# Unveiling the Antioxidant Power and Secondary Metabolites of *Tabebuia chrysantha* (Jacq.) Leaves and Flowers from Ecuador

**DOI:** 10.3390/ph18050649

**Published:** 2025-04-29

**Authors:** Raluca A. Mihai, Ramiro Fernando Vivanco Gonzaga, Nelson Santiago Cubi Insuaste, Nilo Rigoberto Maza Morocho, Rodica D. Catana

**Affiliations:** 1 CIAM, Department of Life Science and Agriculture, Universidad de Las Fuerzas Armadas—ESPE, Av. General Ruminahui s/n y, Sangolqui 171103, Ecuador; 2 Department of Life Science and Agriculture, Universidad de Las Fuerzas Armadas—ESPE, Av. General Ruminahui s/n y, Sangolqui 171103, Ecuador; rfvivanco2@espe.edu.ec (R.F.V.G.); nscubi@espe.edu.ec (N.S.C.I.); 3 IASA 1, Department of Life Science and Agriculture, Universidad de las Fuerzas Armadas—ESPE, Av. General Rumiñahui s/n y Ambato, Sangolquí 171103, Ecuador; nrmaza@espe.edu.ec; 4 Institute of Biology Bucharest of Romanian Academy, 296 Splaiul Independentei, 060031 Bucharest, Romania; rodica.catana@ibiol.ro

**Keywords:** *Tabebuia chrysantha* (Jacq.) Nichols, antioxidant capacity, flavonoids, phenols, phytotherapy, guayacan

## Abstract

**Background**: *Tabebuia chrysantha* (Jacq.) Nichols, commonly known as Guayacan, is a prominent species within the Bignoniaceae family known for its medicinal value and ecological significance. This study aimed to characterize the antioxidant capacity and secondary metabolite composition of Guayacan leaves and flowers grown in Ecuador, a region where its chemical profile remains unexplored. **Methods**: Comprehensive analyses were conducted to determine the total phenolic content (TPC), total flavonoid content (TFC), and antioxidant activity using ABTS, DPPH, FRAP assays, and LC-MS. **Results**: The results revealed remarkable differences between mature leaves and flowers. Leaves exhibited consistently higher flavonoid levels (e.g., 0.280 ± 0.005 mg QE/g DW) and superior antioxidant capacity across all assays (e.g., 10.84 ± 0.51 µmol Trolox g^−^^1^ DW in ABTS) compared to flowers, which showed greater variability but lower overall activity. These findings highlight a functional specialization, with leaves synthesizing more flavonoids to mitigate oxidative stress from environmental factors such as UV radiation. LC-MS analysis unveiled various bioactive compounds, including phenolic acids, flavonoids, and terpenoids. Unique metabolites like α-lipoamide in leaves and oleanolic acid in flowers suggest distinct adaptive roles, potentially linked to stress tolerance and reproductive functions. Additionally, strong correlations among antioxidant assays (e.g., FRAP vs. DPPH, r = 0.993, *p* < 0.001) emphasize the pivotal role of phenolics and flavonoids in free radical scavenging and reduction mechanisms. **Conclusions**: The findings of this study demonstrate the superior antioxidant capacity of leaves, driven by their higher accumulation of flavonoids and phenolic compounds. This research represents a foundational step toward uncovering the therapeutic potential of Ecuadorian Guayacan as a source of natural antioxidants and bioactive compounds, supporting its future applications in phytotherapy and drug development.

## 1. Introduction

The genus *Tabebuia* is the most diverse within the *Bignoniaceae* family, comprising flowering trees with over 100 species distributed across tropical and subtropical regions, extending from the United States to northern Argentina and Chile [1]. *T. chrysantha*, commonly known as the Golden Trumpet Tree, is a medium-sized deciduous tree, reaching heights between 12–22 m, distributed from Mexico to Colombia and Venezuela [2].

In Ecuador, this species is widely found, thriving at altitudes ranging from sea level to 1.500 m above sea level in regions with annual precipitation between 1.000–2.500 mm and temperatures from 12–24 °C [3]. It typically inhabits dry and tropophilous forests, predominantly in the southern regions of Esmeraldas, Manabí, Guayas, and El Oro provinces. The tree is renowned for its striking appearance, characterized by terminal inflorescences of vibrant yellow flowers with a bell-shaped calyx [3,4].

Oxidative stress (an imbalance between free radicals and antioxidants in the body) is a complex process influenced by a combination of internal (e.g., normal cellular processes, body’s immune response to infections or injuries, aging) and external (e.g., environmental pollutants, radiation, smoking, excessive alcohol consumption, poor diet, stress, excessive exercise) factors; it plays a significant role in the development of numerous chronic diseases [5]. Natural products offer a wealth of compounds that can effectively combat oxidative stress, making them invaluable in both prevention and treatment [6].

**Figure 1:**
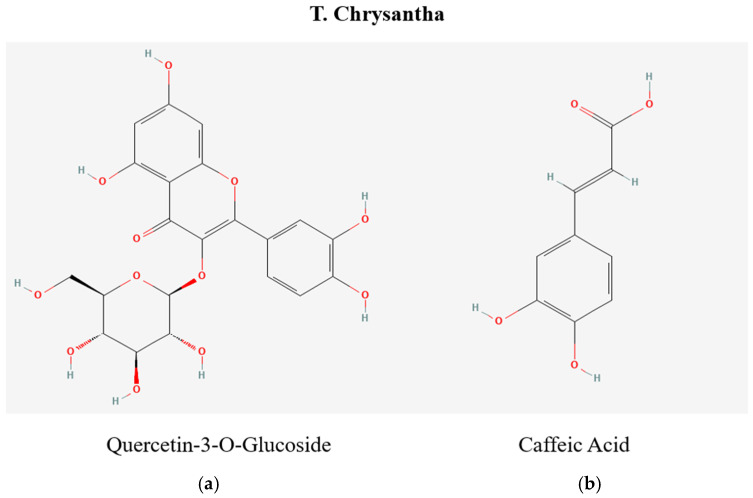
Chemical structures of key bioactive compounds in *T. chrysantha*: (**a**) quercetin-3-O-glucoside, (**b**) caffeic acid.

The genus *Tabebuia* is noted for its diverse array of secondary metabolites, which are active principles in treating various pathologies [7]. *T. chrysantha* shows potential in various pharmacological areas, particularly in its anti-parasitic and antioxidant properties; more research is needed to understand its therapeutic potential fully and to ensure its safe and effective use [8]. Species within this genus are commonly utilized in traditional medicine [9] and are acknowledged as therapeutic alternatives in rural communities. Ethnobotanical and ethnopharmacological studies substantiate these practices, demonstrating their potential to address multiple ailments. This interest has driven the exploration of new phytotherapeutic drugs [10]. The evidence suggests that the *Tabebuia* genus possesses antimicrobial and astringent properties. Moreover, the anti-infective activity of its plant extracts has been evaluated, yielding promising results. *T. chrysantha* and other species of the genus exhibit anti-inflammatory effects in both in vivo and in vitro studies. Ethnopharmacological investigations highlight the potential of *T. chrysantha* in cancer treatment due to its antitumor activity, attributed to compounds such as naphthoquinones and polyphenols [1]. Additionally, the bark contains bioactive compounds, including naphthoquinones, quinones, furanonaphthoquinones, benzoic acid, cyclopentenyl dialdehydes, and flavonoids and other classes of secondary metabolites, including tannins, alkaloids, and iridoids (Figure 1) [9]. Notably, a moderate concentration of flavonoids has been observed in the flowers of *T. chrysantha*.

The importance of this research lies in addressing the current gap in scientific studies on *T. chrysantha* (Golden Trumpet Tree) grown in Ecuador. Despite the abundance of this species in the country, its metabolic profile and biological capacities, including its antioxidant properties, remain unexplored. Evaluating these attributes is crucial, as they hold significant potential in the treatment of various diseases. This study seeks to unveil the therapeutic value of the metabolites found in *T. chrysantha* leaves and flowers. By leveraging the unique biochemical characteristics of Ecuadorian *T. chrysantha*, this work could pave the way for innovative solutions in phytotherapy and drug discovery.

## 2. Results

### 2.1. Bioactive Compound Determination

**Figure 2:**
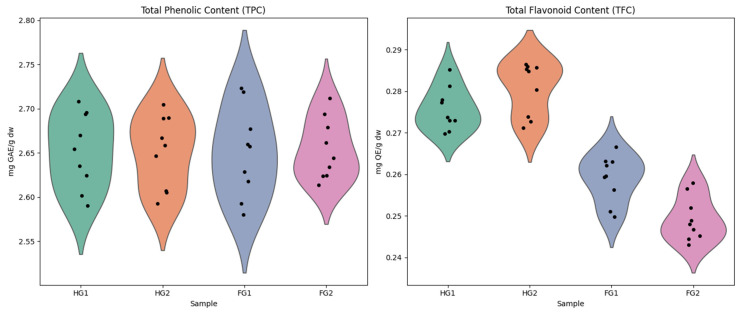
Distribution of total phenolic content (TPC) and total flavonoid content (TFC) in Guayacan leaves and flowers. The violin plots illustrate the variation in TPC (**left**) and TFC (**right**) across four samples: HG1 and HG2 (leaves) and FG1 and FG2 (flowers). Black dots represent individual measurements, providing insight into the variability within each group.

The total phenolic content (TPC) and total flavonoid content (TFC) varied among the samples analyzed of Guayacan leaves and flowers. The TPC values in Guayacan leaves ranged from 2.590 to 2.708 mg GAE/g DW in HG1 (collected from Esmeraldas) and from 2.592 to 2.705 mg GAE/g DW in HG2 (collected from Guayas), with average values of 2.645 ± 0.041 mg GAE/g DW and 2.646 ± 0.042 mg GAE/g DW, respectively. In Guayacan flowers, TPC values ranged from 2.580 to 2.723 mg GAE/g DW in FG1 and from 2.613 to 2.712 mg GAE/g DW in FG2, with averages of 2.653 ± 0.048 mg GAE/g DW and 2.646 ± 0.042 mg GAE/g DW. The TFC values in Guayacan leaves ranged from 0.269 to 0.285 mg QE/g DW in HG1 and from 0.271 to 0.286 mg QE/g DW in HG2, with average values of 0.275 ± 0.005 mg QE/g DW and 0.280 ± 0.005 mg QE/g DW, respectively. In Guayacan flowers, TFC values ranged from 0.249 to 0.266 mg QE/g DW in FG1 and from 0.244 to 0.257 mg QE/g DW in FG2, with averages of 0.260 ± 0.006 mg QE/g DW and 0.249 ± 0.006 mg QE/g DW (Figure 2).

These results indicate that Guayacan leaves generally accumulate higher levels of phenolic and flavonoid compounds compared to Guayacan flowers. The highest TPC value was detected in FG1 (2.723 mg GAE/g DW), whereas the highest TFC was recorded in HG2 (0.286 mg QE/g DW). The observed differences may be attributed to variations in metabolic pathways, environmental factors, or tissue-specific biosynthetic activity. The relatively higher TPC and TFC in leaves suggest a possible role in plant defense mechanisms and adaptation to environmental stress (Table 1).

### 2.2. Antioxidant Capacity Determination

**Figure 3:**
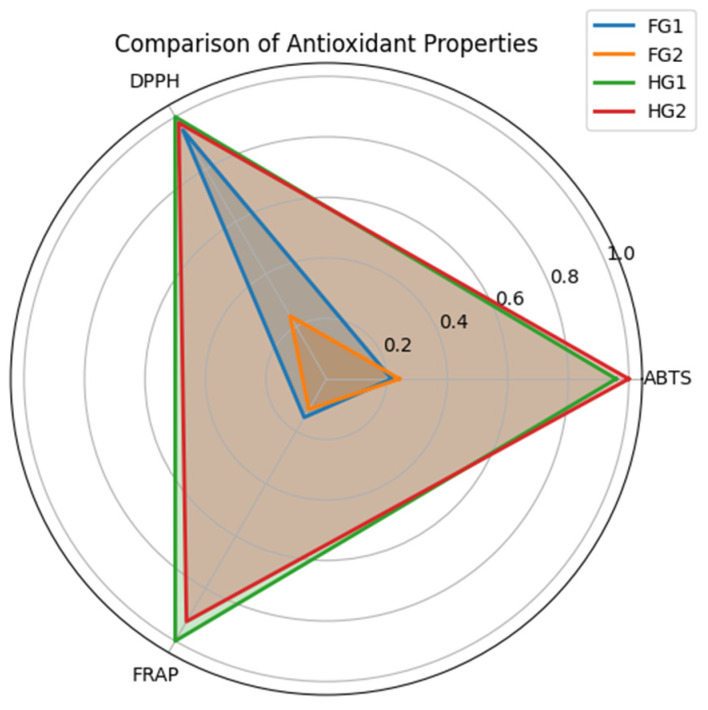
Antioxidant activity determined by FRAP, DPPH, and ABTS assays in Guayacan leaves and flowers. The radar chart represents the average antioxidant activity values for each sample, highlighting significant differences among the methods. Values are based on triplicate replications.

The antioxidant capacity of Guayacan leaves and flowers was evaluated using the ABTS, DPPH, and FRAP methods. The obtained values revealed significant differences among the analyzed samples (Figure 3, Table 1).

For the ABTS assay, Guayacan leaves (HG1 and HG2) exhibited considerably higher antioxidant activity compared to flowers (FG1 and FG2). The average values for HG1 were 10.57 ± 0.76 µmol Trolox g^−^^1^ DW, while HG2 recorded 10.84 ± 0.51 µmol Trolox g^−^^1^ DW. In contrast, the flowers presented significantly lower values, with FG1 reaching 2.32 ± 0.53 µmol Trolox g^−^^1^ DW and FG2 at 2.61 ± 0.06 µmol Trolox g^−^^1^ DW.

The DPPH assay reflected a similar trend, where HG1 exhibited the highest antioxidant capacity with an average value of 37.24 ± 0.89 µmol Trolox g^−^^1^ DW, followed by HG2 with 37.04 ± 1.23 µmol Trolox g^−^^1^ DW. The flowers, however, were discordant in antioxidant activity, as FG1 recorded values comparable to the leaves (35.93 ± 1.36 µmol Trolox g^−^^1^ DW), while FG2 displayed significantly lower values (9.07 ± 0.21 µmol Trolox g^−^^1^ DW).

On the other hand, the reducing activity assessed by FRAP once again demonstrated higher activity in leaves compared to flowers. HG1 had an average of 22.67 ± 0.43 µmol Fe^2^^+^ g^−^^1^ DW, followed by HG2 with 20.88 ± 0.68 µmol Fe^2^^+^ g^−^^1^ DW. Regarding the flowers, FG1 showed intermediate values (3.33 ± 0.14 µmol Fe^2^^+^ g^−^^1^ DW), while FG2 recorded the lowest activity (2.65 ± 0.10 µmol Fe^2^^+^ g^−^^1^ DW).

### 2.3. Correlation Between Bioactive Compounds and Antioxidant Capacity

**Figure 4:**
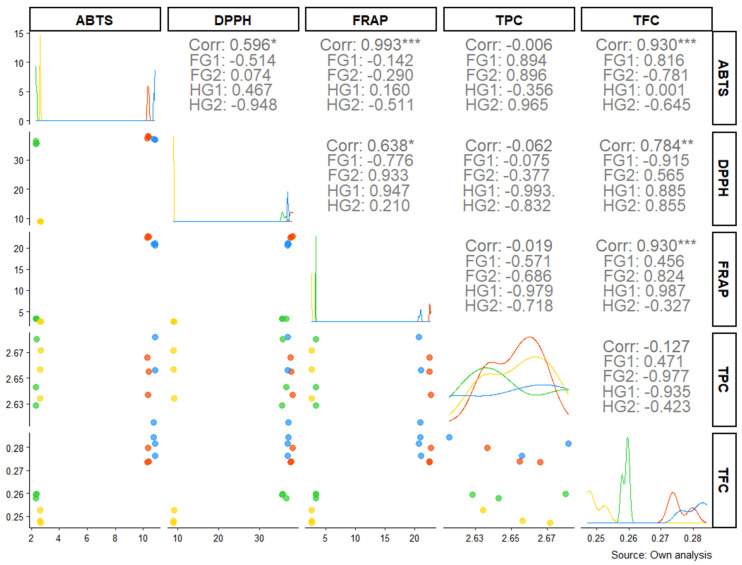
Correlation matrix between antioxidant capacity assays (ABTS, DPPH, and FRAP) and bioactive compounds (TPC and TFC) in Guayacan leaves (HG1 and HG2) and flowers (FG1 and FG2). Correlation coefficients (r) are displayed in each cell, with statistical significance indicated by asterisks (* *p* < 0.05; ** *p* < 0.01; *** *p* < 0.001). The diagonal shows density distributions for each variable, while scatterplots with regression lines illustrate pairwise relationships. The data were analyzed for each tissue type, highlighting differences in the interplay between secondary metabolites and antioxidant properties, different colors indicate data for each sample, lines represent the fitted correlation trend for the respective assays.

The analysis of antioxidant properties in Guayacan leaves (HG1, HG2) and flowers (FG1, FG2) revealed significant correlations among various bioactive compounds and antioxidant assays (ABTS, DPPH, FRAP, TPC, and TFC). These findings highlight the intricate relationships between secondary metabolites and antioxidant capacity across different plant tissues. Strong positive correlations were observed, particularly between FRAP and DPPH (r = 0.993, *p* < 0.001), as well as FRAP and ABTS (r = 0.930, *p* < 0.001), indicating the central role of phenolic compounds in these antioxidant capacities. Similarly, ABTS and TFC showed a strong positive correlation (r = 0.930, *p* < 0.001), suggesting that flavonoids may play an important role in the ABTS antioxidant assay. In contrast, notable negative correlations were identified. For instance, DPPH and ABTS showed a moderate negative correlation (HG2: r = −0.948), suggesting that specific interactions or structural differences in the antioxidant mechanisms might underlie these observations. Furthermore, TPC exhibited weak or negligible correlations with other antioxidant assays, such as FRAP (r = −0.019), highlighting that total phenolic content measured by the TPC assay might not fully explain the antioxidant activity across the tested samples. When comparing plant tissues, the flowers (FG1 and FG2) generally demonstrated stronger positive correlations between antioxidant assays (e.g., FG2: FRAP vs. DPPH, r = 0.933) compared to leaves (HG1 and HG2), where mixed patterns were observed, including strong negative correlations (e.g., HG1: ABTS vs. DPPH, r = −0.776). These variations may reflect differences in the specific composition and concentrations of bioactive compounds between leaves and flowers (Figure 4).

### 2.4. Screening of Bioactive Compounds by Liquid Chromatography Coupled with Mass Spectrometry LC-MS

**Figure 5:**
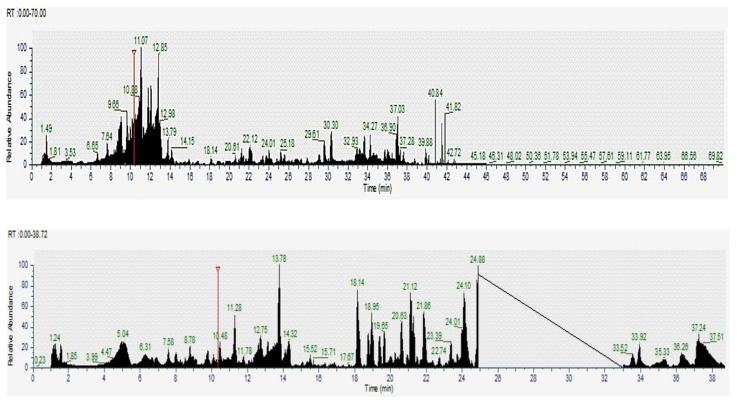
LC-MS chromatogram (positive and negative ion mode) of Guayacan leaves in Guayas. The chromatogram displays the retention times and corresponding molecular ion peaks of various bioactive compounds identified in the sample. Key peaks represent different bioactive metabolites, which contribute to the biochemical profile of the Guayacan leaves. The data obtained from the LC-MS analysis helps to identify compounds associated with the antioxidant and metabolic properties of this plant. Base Peak *m*/*z* = 50.00–2000.00 MS.

**Figure 6:**
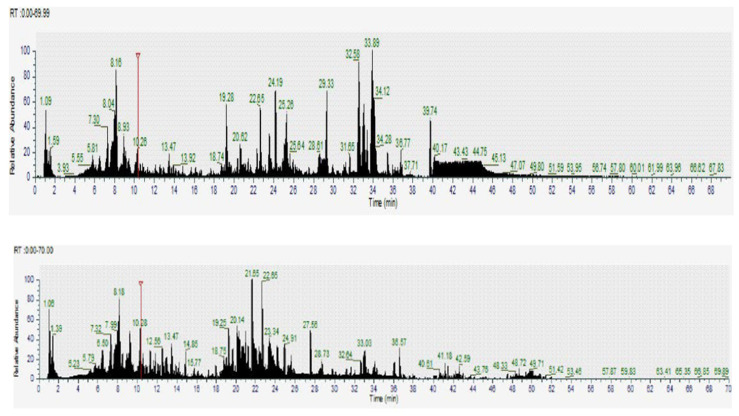
LC-MS chromatogram (positive and negative ion mode) of Guayacan flowers in Guayas. The chromatogram illustrates the retention times and molecular ion peaks of various bioactive compounds identified in the sample. Prominent peaks correspond to flavonoids such as eriodictyol-7-O-glucoside and isorhamnetin-3-O-glucoside, as well as phenolic derivatives like glycyrrhetinic acid and oleanolic acid, which contribute to the antioxidant properties of Guayacan flowers. This profile highlights the richness of secondary metabolites present in this part of the plant. Base Peak *m*/*z* = 50.00–2000.00 MS.

LC-MS was employed to identify bioactive and metabolic compounds in the leaves and flowers of Guayacan, a plant recognized for its potential medicinal properties. The analysis, based on molecular mass and retention times, revealed a wide diversity of significant compounds in both parts of the plant. Several bioactive compounds were identified using positive and negative ionization modes of LC-MS. In the leaves, key compounds such as phenolic acids (e.g., caffeic acid and sinapic acid), flavonoids such as luteolin-8-C-glucoside and quercetin-3-O-β-D-galactopyranoside, and other secondary metabolites such as α-lipoamide and quinurenic acid were highlighted. The flowers, on the other hand, presented a distinctive profile that included eriodictyol-7-O-glucoside, isorhamnetin-3-O-glucoside, and phenolic derivatives such as glycyrrhetinic acid and oleanolic acid (Table 2, Figure 5 and Figure 6).

## 3. Discussion

The analysis of total phenolic content and total flavonoid content in Guayacán leaves and flowers revealed significant tissue-specific variation in the accumulation of secondary metabolites, reflecting the plant’s adaptive strategies to different environmental and physiological demands. Although TPC levels were similar between leaves and flowers, leaves consistently exhibited higher TFC, with values such as 0.280 ± 0.005 mg QE/g DW in HG2 leaves compared to 0.249 ± 0.006 mg QE/g DW in FG2 flowers. This suggests a functional specialization in leaves, where flavonoid biosynthesis is prioritized due to their role as antioxidants and photoprotectors against environmental stresses such as UV radiation [11,12]. In contrast, flowers, despite having comparable TPC levels, may allocate phenolic resources to compounds involved in reproductive functions, such as pollinator attraction or floral pathogen defense [13]. The slight variation among samples (HG1, HG2, FG1, and FG2) highlights the influence of factors such as microclimatic conditions or phenological timing on secondary metabolite profiles [14]. These results align with studies emphasizing the need for multiple replicates to capture plant chemical diversity [14], underscoring the robustness of our experimental design.

The antioxidant capacity, assessed through ABTS, DPPH, and FRAP assays, demonstrated the superiority of leaves over flowers, with marked differences in their ability to mitigate oxidative stress. In the ABTS assay, leaves showed significantly higher activity (e.g., 10.57 ± 0.76 µmol Trolox/g DW in HG1) compared to flowers (e.g., 2.32 ± 0.53 µmol Trolox/g DW in FG1). This trend was reflected in the FRAP assay, where leaves exhibited strong reducing power (e.g., 22.67 ± 0.43 µmol Fe^2^^+^/g DW in HG1) compared to flowers (e.g., 3.33 ± 0.14 µmol Fe^2^^+^/g DW in FG1). These findings are consistent with the high flavonoid content in leaves, known for neutralizing reactive oxygen species (ROS) generated during photosynthesis [15]. The greater antioxidant capacity in leaves likely serves as a protective mechanism against environmental stressors such as solar radiation and herbivory [10], whereas the lower activity in flowers may reflect their transient nature and distinct metabolic priorities during reproduction [13]. This pattern reinforces the hypothesis of functional specialization between tissues in Guayacán.

The DPPH assay provided additional insights, revealing not only the superiority of leaves but also notable variability among flower samples. Leaves exhibited high antioxidant capacity, with values such as 37.24 ± 0.89 µmol Trolox/g DW in HG1 and 37.04 ± 1.23 µmol Trolox/g DW in HG2, while flowers showed divergent behavior: FG1 recorded 35.93 ± 1.36 µmol Trolox/g DW, comparable to leaves, whereas FG2 had only 9.07 ± 0.21 µmol Trolox/g DW. This discrepancy suggests that factors such as developmental stage or microenvironmental conditions influence antioxidant accumulation in floral tissues [13]. For instance, flowers at different maturity stages or those exposed to biotic stress may exhibit altered secondary metabolite profiles, as observed in other species [13]. The high DPPH value in FG1 indicates that, under certain conditions, Guayacán flowers can accumulate significant levels of bioactive compounds, warranting further investigation into the modulators of this variability.

Comparatively, the antioxidant capacity of Guayacán leaves rivals that of other well-studied natural sources. For example, the DPPH activity in HG1 leaves (37.24 ± 0.89 µmol Trolox/g DW) is similar to that reported for guava extracts (~30–40 µmol Trolox/g DW), a fruit recognized for its antioxidant properties. This positions Guayacán leaves as promising candidates for applications in functional foods or pharmaceuticals, where natural antioxidants are valued for their potential to mitigate oxidative stress-related disorders [16]. The consistent performance of leaves across multiple antioxidant assays underscores their reliability as a source of bioactive compounds, in contrast to the more variable profile of flowers. This comparison contextualizes the results and opens the door to exploring the economic and ecological value of Guayacán across different sectors.

The correlation analysis between antioxidant assays and bioactive compounds provided deeper insights into the underlying mechanisms. Strong positive correlations were observed between FRAP and DPPH (r = 0.993, *p* < 0.001) and between FRAP and ABTS (r = 0.930, *p* < 0.001), suggesting that phenolic compounds play a central role in free radical scavenging and reducing activities [17]. The positive correlation between ABTS and TFC (r = 0.930, *p* < 0.001) highlights the contribution of flavonoids to ABTS radical neutralization, likely due to their catechol groups and conjugated double bonds [18]. These findings reinforce the importance of employing multiple assays to capture diverse antioxidant mechanisms [18], validating the methodological robustness of this study and providing a solid foundation for future research on the antioxidant chemistry of Guayacán.

However, this study also revealed negative correlations and tissue-specific patterns that merit further exploration. A moderate negative correlation was observed between DPPH and ABTS in HG2 leaves (r = −0.948), possibly reflecting differential sensitivities of these assays to various antioxidant classes. While DPPH is more sensitive to lipophilic antioxidants, ABTS detects both hydrophilic and lipophilic compounds [19], which may explain the discrepancies. Additionally, the weak correlation between TPC and FRAP (r = −0.019) suggests that total phenolic content, as measured by the Folin–Ciocalteu method, does not fully explain the antioxidant activity [20]. This may be attributed to non-phenolic reducing agents or phenols with different reactivities [20], highlighting the complexity of plant antioxidant systems and the need for targeted analyses in future studies.

Tissue-specific differences in correlation patterns were also evident. Flowers (FG1 and FG2) showed consistently positive correlations between antioxidant assays (e.g., FRAP vs. DPPH, r = 0.933 in FG2), whereas leaves (HG1 and HG2) exhibited mixed results, including negative correlations (e.g., ABTS vs. DPPH, r = −0.776 in HG1).These disparities reflect variations in secondary metabolite composition between tissues [21,22]. Flowers may be enriched in specific flavonoids or phenolic acids that uniformly enhance antioxidant activity [21], whereas the more diverse profile of leaves, shaped by their roles in photosynthesis and defense, could result in more complex antioxidant interactions [22]. This analysis underscores the importance of considering physiological differences between tissues when interpreting antioxidant data.

The LC-MS analysis identified a diversity of secondary metabolites in Guayacán leaves and flowers, including bioactive compounds with known antioxidant properties. Flavonoids such as quercetin-3-O-glucoside and luteolin-8-C-glucoside, along with phenolic acids such as caffeic acid, were detected in both tissues, aligning with the metabolic profiles of Bignoniaceae [23,24]. These compounds are recognized for scavenging free radicals and mitigating oxidative stress [18,19], supporting the observed antioxidant potential. The presence of unique compounds—such as α-lipoamide in leaves and oleanolic acid in flowers—suggests chemical specialization [25,26]. α-Lipoamide may be linked to stress adaptation in leaves [25], while oleanolic acid in flowers could contribute to anti-inflammatory defenses [26]. These findings highlight the ecological and physiological importance of secondary metabolites in Guayacán, justifying future studies on their roles and applications.

## 4. Materials and Methods

### 4.1. Sample Collection

Mature leaves and flowers of Guayacan were collected in Esmeraldas (HG1 and FG1) and Guayas (HG2 and FG2) provinces to capture the diversity of environmental conditions in March 2023, during the dry season. Ten specimens were collected per site (5 leaves, 5 flowers), Flowers were collected at the full bloom stage to standardize the samples. The two provinces (Esmeraldas and Guayas) were chosen because of their differences in precipitation (Esmeraldas: ~1500 mm/year, Guayas: ~1000 mm/year) and mean temperature (Esmeraldas: 25–27 °C, Guayas: 23–25 °C), allowing the evaluation of environmental influences. These sites were strategically chosen to evaluate the potential influence of climatic and altitudinal variations on the antioxidant properties and secondary metabolite composition of Guayacan leaves and flowers.

### 4.2. Extraction of Bioactive Compounds

The extraction of bioactive compounds was performed following the protocol described by Claros (2021) [27] with modifications to optimize the process for Guayacan leaves and flowers. Fresh, mature plant material was finely ground using a mortar and pestle to obtain a homogeneous powder. Exactly 1 g of the powdered sample was weighed using an analytical balance and subsequently macerated in 15 mL Falcon tubes with 96% ethanol (10 mL), stirred with a glass rod, and kept for 72 h at 5 °C. Extractions were performed in triplicate; absorbance was measured with a UV–Vis spectrophotometer. Extracts were analyzed at a fixed concentration (1 g/10 mL ethanol) to establish a baseline. Future studies will incorporate dilution series to evaluate concentration-dependent effects. Analyses were performed with a UV–Vis spectrophotometer (Thermo Scientific Genesys 10S, Waltham, MA, USA), LC-MS (Vanquish HPLC, Thermo Fisher Scientific), and reagents from Sigma–Aldrich (Trolox, FeSO_4_-7H_2_O, DPPH, ABTS, AlCl_3_, and CH_3_COONa) (St. Louis, MO, USA).

### 4.3. Determination of Active Ingredients

The Folin–Ciocalteu colorimetric method (López-Froilán et al. (2018), [28]) with modifications, was used to determine the total phenolic content of Guayacan samples (leaves and flowers) by mixing the ethanolic extracts (0.4 mL) with Folin–Ciocalteu reagent diluted to 10% (*v*/*v*) (2 mL) and 7.5% sodium carbonate (1.6 mL). The absorbance of the mixture incubated at room temperature for 30 min was read at 765 nm. The calibration curve was performed using gallic acid solutions (0–250 mg/L), the blank being prepared by replacing the sample with ethanol. The results are expressed as milligrams of gallic acid equivalents per liter (mg GAE/L). For the determination of flavonoid content, the colorimetric aluminum chloride test was used, as described by Pekal et al. (2014) [29]. Ethanolic extracts (1 mL) were combined with 1.5 mL of solvent, 100 µL of 1 M sodium acetate (CH_3_COONa), 100 µL of 10% (*v*/*v*) aluminum chloride (AlCl_3_), and 2.3 mL of distilled water. The mixtures were incubated at room temperature for 40 min, after which their absorbance was recorded at 435 nm. A calibration curve was generated using quercetin solutions (0–1.5 mg/L), and the results were expressed as milligrams of quercetin equivalents per liter (mg QE/L).

### 4.4. Evaluation of Antioxidant Capacity Using FRAP, DPPH, and ABTS Assays

The antioxidant capacity of Guayacan leaves and flowers was evaluated using three complementary assays: FRAP, DPPH, and ABTS. These complementary methods provided a comprehensive assessment of the antioxidant potential of Guayacan leaves and flowers, elucidating their ability to scavenge free radicals and reduce metal ions under different conditions. All assays were conducted in triplicate for all samples (mature leaves and flowers) collected from two sites (Esmeraldas and Guayas).

The FRAP assay, which quantifies the reduction of Fe^3^^+^ to Fe^2^^+^, was conducted following the methodology described by Rajurkar et al. (2011) [30] with modifications. The FRAP reagent was prepared using 300 mM acetate buffer (pH 3.6), 40 mM HCl, and 20 mM FeCl_3_·6H_2_O. For the analysis, 100 µL of the plant extract was mixed with 300 µL of distilled water and 3 mL of the FRAP solution and incubated for 30 min at room temperature. The absorbance was measured at 593 nm. The calibration curve was constructed by using FeSO_4_ · 7H_2_O (0–5 mM). The results are expressed as Fe^2^^+^ equivalents.

The DPPH assay, used to determine the radical scavenging activity of antioxidants, was performed according to the method of Sachett et al. (2021) [31] and Thaweesang (2019) [32], with modifications. A DPPH stock solution (1 µg L^−^^1^) was prepared, and 2 mL of this solution was mixed with 0.1 mL of plant extract. The reaction mixture was incubated in the dark at room temperature for 30 min, and absorbance was recorded at 517 nm. The percentage of radical scavenging activity was calculated using a standard curve, with Trolox as a reference compound.

The method of Kuskoski et al. (2005) [33] was used for the ABTS assay. In this method, the ABTS•+ radical is generated and adjusted to an absorbance of 0.7 ± 0.1 at 754 nm. The mixture (ABTS solution (2 mL) and extract (20 µL)) was incubated for 7 min in the dark. The absorbance was measured at 754 nm, and the results are expressed in Trolox equivalents (TE).

### 4.5. Determination of Bioactive Compounds by LC-MS

Liquid chromatography–mass spectrometry (LC-MS) was used to identify bioactive compounds from Guayacan samples (leaves and flowers) collected from Guayas according to the methodology described by Tohma et al. (2016) [34], with modifications. Extracts were produced using 20 mL of 80% ethanol starting from 1 g of lyophilized plant material; subsequently, the extract was incubated at 30 °C for 2 h [35]. Samples were centrifuged (5000 rpm) for 10 min at 4 °C, filtered, and the solvent was removed by rotary evaporation at 30 °C. The extracts thus obtained were stored at −20 °C in airtight tubes.

The LC-MS system used for the analysis consisted of a Vanquish HPLC unit (Thermo Fisher Scientific) coupled to an Ion Trap mass spectrometer. Chromatographic separation was performed using an Accucore Vanquish column (150 × 2.1 mm) maintained at 35 °C, with a flow rate of 0.5 mL/min [36]. A 10 µL injection of 0.1% formic acid solution was employed as the mobile phase. The identification of bioactive compounds was achieved by comparing mass spectra and retention times with standard ions from reference databases such as PubChem, ChEBI, Metlin, and HPLC repositories. Data processing and metabolite identification were conducted using MZmine 2.53 software, complemented with information from the scientific literature [37].

### 4.6. Statistical Analysis

Statistical analyses were performed using RStudio software (R version 4.3.2). A two-factor ANOVA was conducted to determine significant differences among groups, with a significance threshold set at *p* < 0.05. All experiments (TPC, TFC, ABTS, DPPH, and FRAP) were performed in triplicate for each sample (HG1, HG2, FG1, and FG2), with results expressed as mean ± standard deviation (n = 3 per sample, total n = 12 per essay). Additionally, Pearson’s correlation coefficient was calculated to assess the relationship between secondary metabolite content and antioxidant capacity.

## 5. Conclusions

This study highlights the significant role of *Tabebuia chrysantha* (Guayacan) as a source of natural antioxidants and bioactive compounds, particularly in leaves and flowers grown in Ecuador. The findings demonstrate the superior antioxidant capacity of leaves, driven by their higher accumulation of flavonoids and phenolic compounds, which are essential for mitigating oxidative stress caused by environmental factors like UV radiation. Flowers, while exhibiting lower antioxidant activity on average, revealed variability that could be influenced by factors such as growth conditions, developmental stages, or genetic variability. This underscores the importance of tissue-specific adaptations and their functional roles in the plant’s metabolism.

The LC-MS analysis further confirmed the presence of diverse secondary metabolites, including phenolic acids, flavonoids, and terpenoids, which exhibit strong antioxidant properties. The detection of unique compounds, such as α-lipoamide in leaves and oleanolic acid in flowers, highlights their potential as valuable bioactive resources for applications in phytotherapy and drug development. The strong correlations between antioxidant assays and bioactive compounds emphasize the importance of phenolics and flavonoids in free radical scavenging mechanisms, although the observed discrepancies point to the complexity of interactions among different metabolites.

In conclusion, this research established a foundational understanding of the chemical and biological potential of *T. chrysantha* cultivated in Ecuador, a species previously understudied in this region. By unveiling its antioxidant properties and metabolic diversity, this study not only highlights its therapeutic potential but also paves the way for further exploration into its application in pharmacology. These findings reinforce the need for continued research into Ecuadorian flora as a source of innovative solutions for health and wellness challenges.

## Figures and Tables

**Table 1:** Summary of Total Phenol Content (TPC), Total Flavonoids (TFC) and Antioxidant Capacity in Guaiacum Leaves and Flowers.

Samples	TPC (mg GAE/g DW)	TFC (mg QE/g DW)	ABTS (µmol Trolox/g DW)	DPPH (µmol Trolox/g DW)	FRAP (µmol Fe^2^^+^/g DW)
HG1	2.645 ± 0.041	0.275 ± 0.005	10.57 ± 0.76	37.24 ± 0.89	22.67 ± 0.43
HG2	2.646 ± 0.042	0.280 ± 0.005	10.84 ± 0.51	37.04 ± 1.23	20.88 ± 0.68
FG1	2.653 ± 0.048	0.260 ± 0.006	2.32 ± 0.53	35.93 ± 1.36	3.33 ± 0.14
FG2	2.646 ± 0.042	0.249 ± 0.006	2.61 ± 0.06	9.07 ± 0.21	2.65 ± 0.10

Note: Values expressed as mean ± standard deviation (n = 3).

**Table 2:** Bioactive and Metabolic Compounds tentatively assigned in Guayacan Leaves and Flowers Using LC-MS.

ID	*m*/*z*	Retention Time	Proposed Compound Identity	Molecular Ion	Molecular Formula	Plant Source	Ionization Mode
2057	465.307	1511	Quercetin-3-O-glucoside (Hyperoside)	M+H	C2_1_H_20_O_12_	Flower and Leaf	Positive
3915	449.276	7392	Luteolin-8-C-glucoside	M+H	C_21_H_20_O_11_	Flower and Leaf	Positive
8557	465.351	11,135	Quercetin-3-O-β-D-galactopyranoside	M+H	C_21_H_20_O_12_	Flower and Leaf	Positive
11474	419.357	13,177	Liquiritin	M+H	C2_1_H_22_O_9_	Flower and Leaf	Positive
13457	593.187	14,666	Acacetin-7-O-rhamnosylglucoside (Fortunellin)	M+H	C_28_H_32_O_14_	Flower and Leaf	Positive
1820	179.188	19.878	Caffeic acid *	M-H	C_9_H_8_O_4_	Flower and Leaf	Negative
2443	223.233	24.767	Sinapinic acid	M-H	C_11_H_12_O_5_	Flower and Leaf	Negative
1966	340.046	21.087	Aristolochic acid C	M-H	C_17_H_11_NO_7_	Flower and Leaf	Negative
1242	375.129	15.621	Deoxyloganic acid	M-H	C_16_H_24_O_10_	Flower and Leaf	Negative
2136	206.169	1623	α-Lipoamide	M+H	C_8_H_15_NOS_2_	Leaf	Positive
2446	190.135	3799	Kynurenic acid	M+H	C_10_H_7_NO_3_	Leaf	Positive
3159	419.286	5999	Aloin A	M+H	C_21_H_22_O_9_	Leaf	Positive
4606	463.088	8073	Kaempferol-3-O-glucuronide	M+H	C_21_H_18_O_12_	Leaf	Positive
6315	449.320	9576	Plantaginin	M+H	C_21_H_20_O_11_	Leaf	Positive
6331	481.365	9653	3,5,7,8,3,4-Hexahydroxyflavone-8-O-glucoside	M+H	C_21_H_20_O_13_	Leaf	Positive
7746	519.389	10.51	6-O-Malonylcosmosiin	M+H	C_24_H_22_O_14_	Leaf	Positive
10032	479.398	12,051	Isorhamnetin-3-O-glucoside	M+H	C_22_H_22_O_12_	Leaf	Positive
10052	315.316	12.09	Velutin	M+H	C_16_H_12_O_6_	Leaf	Positive
10463	257.284	12,389	Pinocembrin	M+H	C_15_H_12_O_4_	Leaf	Positive
10896	241.265	12,693	6-Hydroxyflavanone	M+H	C_15_H_12_O_3_	Leaf	Positive
11006	293.368	12,932	(10E,15E)-9,12,13-Trihydroxyoctadeca-10,15-dienoic acid	M-2H_2_O+H	C_18_H_28_O_3_	Leaf	Positive
12854	623.430	14.12	Pectolinarin	M+H	C_29_H_34_O_15_	Leaf	Positive
19210	449.108	24,978	Luteolin-6-C-glucoside	M+	C_21_H_20_O_11_	Leaf	Positive
1996	205.097	1472	L-Tryptophan	M+H	C_11_H_12_N_2_O_2_	Leaf	Positive
3409	225.184	6465	Phenazine-1-carboxylic acid	M+H	C_13_H_8_N_2_O_2_	Leaf	Positive
18477	329.309	22,338	Labetalol	M+H	C_19_H_24_N_2_O_3_	Leaf	Positive
18850	255.316	23,717	10,11-Dihydro-10-hydroxycarbamazepine	M+H	C_15_H_14_N_2_O_2_	Leaf	Positive
321	181.254	5.192	Sorbitol	M-H	C_6_H_14_O_6_	Leaf	Negative
566	179.231	5.365	Hexose (e.g., glucose, fructose, mannose, galactose)	M-H	C_6_H_12_O_6_	Leaf	Negative
1961	190.281	21.053	5-Hydroxyindoleacetic acid (5-HIAA)	M-H	C_10_H_9_NO_3_	Leaf	Negative
9962	451.404	12,045	Eriodictyol-7-O-glucoside	M+H	C_21_H_22_O_11_	Flower	Positive
10032	479.398	12,051	Isorhamnetin-3-O-glucoside	M+H	C_22_H_22_O_12_	Flower	Positive
17819	415.396	2051	Chafuroside A	M+H	C_21_H_20_O_11_	Flower	Positive
18383	427.292	21,962	Leupeptin	M+H	C_20_H_38_N_6_O_4_	Flower	Positive
18391	291.311	21,978	Catechin	M+H	C_15_H_14_O_6_	Flower	Positive
18569	299.313	22,705	Enterolactone	M+H	C_18_H_18_O_4_	Flower	Positive
18887	471.447	23,737	Glycyrrhetinic acid	M+	C_30_H_46_O_4_	Flower	Positive
19210	281.303	24,938	Aspartylphenylalanine	M+H	C_13_H_16_N_2_O_5_	Flower	Positive
19406	299.295	25,884	Enterolactone	M+H	C_18_H_18_O_4_	Flower	Positive
19422	365.417	25,948	Xanthosine 5′-monophosphate (XMP)	M+H	C_10_H_13_N_4_O_9_P	Flower	Positive
19630	427.472	2698	Leupeptin	M+	C_20_H_38_N_6_O_4_	Flower	Positive
20879	265.297	30,936	Abscisic acid	M+H	C_15_H_20_O_4_	Flower	Positive
20907	323.352	30,936	Chloramphenicol	M+	C_11_H_12_C_l2_N_2_O_5_	Flower	Positive
21401	277.338	32,298	Glutamylglutamic acid	M+H	C_10_H_16_N_2_O_7_	Flower	Positive
21903	415.476	33,398	Podophyllotoxin	M+H	C_22_H_22_O_8_	Flower	Positive
23063	609.531	35,284	3,10S-Dihydroxypheophorbide	M+H	C_33_H_34_N_4_O_6_	Flower	Positive
23713	457.513	36,385	Oleanolic acid	M+H	C_30_H_48_O_3_	Flower	Positive

Legend: Compounds tentatively identified by a comparison of fragmentation and formulas with databases (PubChem, Metlin) and the literature. *m*/*z* observed to three decimal places; *m*/*z* calculated according to exact masses in [M+H]^+^ or [M-H]-mode. Tolerance: ±0.001 Da. Retention in minutes. *—previously mentioned in the literature for the same species.

## Data Availability

The original contributions presented in this study are included in the article; further inquiries can be directed to the corresponding author due to privacy.

## References

[1] Panda S.P., Panigrahy U.P., Panda S., Jena B.R. (2019). Stem extract of *Tabebuia chrysantha* induces apoptosis by targeting sEGFR in Ehrlich Ascites Carcinoma. J. Ethnopharmacol..

[2] Döring M. (2023). GBIF Backbone Patch. GBIF Secretariat. Checklist Dataset.

[3] Vinueza M. *Tabebuia chrysantha* (Jacq.) Nicholson. 201). https://ecuadorforestal.org/fichas-tecnicas-de-especies-forestales/ficha-tecnica-no-6-guayacan.

[4] Rojas-Rodríguez F., Torres-Córdoba G. (2016). Árboles del Valle Central de Costa Rica: Reproducción cortés amarillo *Tabebuia chrysantha* (Jacq.) Nichols. Revista Forestal Mesoamericana Kurú.

[5] Block G., Dietrich M., Norkus E.P., Morrow J.D., Hudes M., Caan B., Packer L. (2022). Factors Associated with Oxidative Stress in Human Populations. Am. J. Epidemiol..

[6] Pizzino G., Irrera N., Cucinotta M., Pallio G., Mannino F., Arcoraci V., Squadrito F., Altavilla D., Bitto A. (2017). Oxidative Stress: Harms and Benefits for Human Health. Oxid. Med. Cell Longev..

[7] Martínez J., Sierra J., Arrubla R., Martínez P. (2011). Metabolitos secundarios en el guayacán amarillo y en el guayacán. Scientia et Technica.

[8] Cardona-Trujillo M.C., Jiménez-González F.J., Veloza L.A., Sepúlveda-Arias J.C. (2024). In Vitro Anti-*Toxoplasma* Activity of Extracts Obtained from *Tabebuia rosea* and *Tabebuia chrysantha*: The Role of β-Amyrin. Molecules.

[9] El-Hawary S.S., Taher M.A., Saleh E., AbouZid S.F., Mohammed R. (2021). Genus Tabebuia: A comprehensive review journey from past achievements to future perspectives. Arabian J. Chem..

[10] Jiménez F.V.L., Sepúlveda J. (2013). Anti-infectious activity in plants of the genus Tabebuia. Scientiarum.

[11] Fini A., Brunetti C., Di Ferdinando M., Ferrini F., Tattini M. (2011). Stress-induced flavonoid biosynthesis and the antioxidant machinery of plants. Plant Sign Behav..

[12] Tattini M., Loreto F., Fini A., Guidi L., Brunetti C. (2015). Isoprenoids and phenylpropanoids are part of the antioxidant defense orchestrated daily by drought-stressed *Platanus × acerifolia* leaves during Mediterranean summers. New Phytol..

[13] Schiestl F.P., Johnson S.D. (2013). Pollinator-mediated evolution of floral signals. Trends Ecol. Evol..

[14] Zargoosh Z., Ghavam M., Tavili A. (2023). Environmental factors affecting the phenolic content and antioxidant activity of medicinal plants: A review. Ind. Crops Product..

[15] Agati G., Brunetti C., Fini A., Gori A., Guidi L., Landi M., Sebastiani F., Tattini M. (2020). Are flavonoids sensors of environmental changes? A review on their biosynthesis and functions in plants under abiotic stress. Plant Cell Environ..

[16] Kumar S., Pandey A.K. (2013). Chemistry and biological activities of flavonoids: An overview. Sci. World J..

[17] Thaipong K., Boonprakob U., Crosby K., Cisneros-Zevallos L., Byrne D.H. (2006). Comparison of ABTS, DPPH, FRAP, and ORAC assays for estimating antioxidant activity from guava fruit extracts. J. Food Compos. Anal..

[18] Pietta P.G. (2000). Flavonoids as antioxidants. J. Nat. Prod..

[19] Huang D., Ou B., Prior R.L. (2005). The chemistry behind antioxidant capacity assays. J. Agric. Food Chem..

[20] Ksouri R., Megdiche W., Falleh H., Trabelsi N., Boulaaba M., Smaoui A., Abdelly C. (2008). Influence of biological, environmental and technical factors on phenolic content and antioxidant activities of Tunisian halophytes. C. R. Biol..

[21] Wojdyło A., Oszmiański J., Czemerys R. (2007). Antioxidant activity and phenolic compounds in 32 selected herbs. Food Chem..

[22] Wang W., Sun C., Mao L., Ma P., Liu F., Yang J., Gao Y. (2016). The biological activities, chemical stability, metabolism and delivery systems of quercetin: A review. Trends Food Sci. Technol..

[23] Martins F.O., Esteves P.F., Mendes G.S., Barbi N.S., Menezes F.S. (2018). Phytochemical and pharmacological overview of Bignoniaceae family. J. Pharm. Pharmacol..

[24] Ferraz-Filha Z.S., Araújo M.C.D.P.M., Ferrari F.C., Dutra I.P.A.R. (2016). *Tabebuia roseoalba*: In vivo hypouricemic and anti-inflammatory effects of its ethanolic extract and constituents. Planta Med..

[25] Packer L., Cadenas E. (2019). Lipoic acid: Energy metabolism and redox regulation of transcription and cell signaling. J. Clin. Biochem. Nutr..

[26] Ayeleso T.B., Matumba M.G., Mukwevho E. (2017). Oleanolic acid and its derivatives: Biological activities and therapeutic potential in chronic diseases. Molecules.

[27] Claros P. (2021). Evaluacion de la Capacidad Antioxidante Total y Contenido de Polifenoles Totales del Phaseolus vulgaris “Frijol”.

[28] López-Froilán R., Hernández-Ledesma B., Cámara M., Pérez-Rodríguez M. (2018). Evaluation of the Antioxidant Potential of Mixed Fruit-Based Beverages: A New Insight on the Folin-Ciocalteu Method. Food Anal. Met..

[29] Pekal A., Pyrzynska K. (2014). Evaluation of aluminum complexation reaction for flavonoid content assay. Food Anal. Met..

[30] Rajurkar N.S., Hande S.M. (2011). Estimation of phytochemical content and antioxidant activity of some selected traditional. Indian. J. Pharm. Sci..

[31] Sachett A., Gallas-Lopes M., Conterato G.M.M., Herrmann A., Piato A. (2021). Antioxidant Activity by DPPH Assay: In Vitro Protocol. Protocols Io. https://www.protocols.io/view/antioxidant-activity-by-dpph-assay-in-vitro-protocbtbpnimn.

[32] Thaweesang S. (2019). Antioxidant activity and total phenolic compounds of fresh and blanching banana blossom (*Musa* ABB CV. Kluai “Namwa”) in Thailand. IOP Conf. Series Mater. Sci. Eng..

[33] Kuskoski E.M., Asuero A.G., Troncoso A.M., Mancini-Filho J., Fett R. (2005). Aplicación de diversosmétodos químicos para determiner actividad antioxidante en pulpa de frutos. Food Sci. Technol..

[34] Tohma H., Koksal E., Kılıc O., Alan Y., Yılmaz M.A., Gulcin I., Bursal E., Alwasel S.H. (2016). RP-HPLC/MS/MS analysis of the phenolic compounds, antioxidant and antimicrobial activities of *Salvia* L. species. Antioxidants.

[35] Irakli M., Skendi A., Bouloumpasi E., Chatzopoulou P., Biliaderis C.G. (2021). LC-MS identification and quantification of phenolic compounds in solid residues from the essential oil industry. Antioxidants.

[36] Kluskal T., Castillo S., Villar-Briones A., Orešič M. (2010). MZmine 2: Modular framework for processing, visualizing, and analyzing mass spectrometry-based molecular profile data. BMC Bioinform..

[37] Cellier G., Moreau A., Chabirand A., Hostachy B., Ailloud F., Prior P. (2015). A Duplex PCR Assay for the Detection of *Ralstonia solanacearum* Phylotype II Strains in *Musa* spp.. PLoS ONE.

